# High-throughput screening carbon and nitrogen sources to promote growth and sporulation in *Rhizopus arrhizus*

**DOI:** 10.1186/s13568-024-01733-0

**Published:** 2024-06-28

**Authors:** Heng Zhao, Xiao Ju, Yong Nie, Timothy Y. James, Xiao-Yong Liu

**Affiliations:** 1 College of Life Sciences, Shandong Normal University, Jinan, 250358 China; 2 State Key Laboratory of Efficient Production of Forest Resources, School of Ecology and Nature Conservation, Beijing Forestry University, Beijing, 100083 China; 3 State Key Laboratory of Mycology, Institute of Microbiology, Chinese Academy of Sciences, Beijing, 100101 China; 4 Graduate School, China Pharmaceutical University, Nanjing, 211198 China; 5 School of Civil Engineering and Architecture, Anhui University of Technology, Ma’anshan, 243002 China; 6 Department of Ecology and Evolutionary Biology, University of Michigan, Ann Arbor, MI 48109-1048 USA

**Keywords:** *Rhizopus oryzae*, *Rhizopus delemar*, Fungal physiology, Fungal phenotype, Biolog FF MicroPlate

## Abstract

*Rhizopus arrhizus* is a saprotrophic, sometimes clinically- and industrially-relevant mold (*Mucorales*) and distributed worldwide, suggesting it can assimilate a broad spectrum of substrates. Here, 69 strains of *R. arrhizus* were investigated by using the Biolog FF MicroPlate for the profiles of utilizing 95 carbon and nitrogen substrates. The study showed that most *R. arrhizus* strains were similar in average well color development (AWCD) and substrate richness (SR). Nevertheless, 13 strains were unique in principal component analyses, heatmap, AWCD, and SR analyses, which may imply a niche differentiation within *R. arrhizus*. The species *R. arrhizus* was able to utilize all the 95 carbon and nitrogen substrates, consistent with the hypothesis of a great metabolic diversity. It possessed a substrate preference of alcohols, and seven substrates were most frequently utilized, with *N*-acetyl-d-galactosamine and l-phenylalanine ranking at the top of the list. Eight substrates, especially l-arabinose and xylitol, were capable of promoting sporulation and being applied for rejuvenating degenerated strains. By phenotyping *R. arrhizus* strains in carbon and nitrogen assimilation capacity, this study revealed the extent of intra-specific variability and laid a foundation for estimating optimum substrates that may be useful for industrial applications.

**Supplementary Information:**

The online version contains supplementary material available at 10.1186/s13568-024-01733-0.

## Introduction

*Rhizopus arrhizus* (*Fungi*, *Mucoromycota*, *Mucoromycetes*, *Mucorales*, and *Rhizopodaceae*) was proposed by Alfred Fischer in 1892, synonymizing *Rhizopus oryzae* Went and Prins. Geerl. 1895. The species is characterized by sparse rhizoids, apophysate sporangia containing small and regular sporangiospores, and sporangiophores forming on stolons at the same point as rhizoids but in opposite direction (Zheng et al. 2007; Zhao et al. 2023). The *R. arrhizus* species are classified into three varieties, namely var. *arrhizus*, var. *tonkinensis*, and var. *delemar*, based on morphological and molecular phylogenetic evidence (Zheng et al. 2007; Liu et al. 2008; Dolatabadi et al. 2014), which is supported by biochemistry and physiology (Londoño-Hernández et al. 2017; Saito et al. 2004; Yao et al. 2018). All these suggest that *R. arrhizus* has a phylogenetic and genetic complexity, and a morphological diversity. A comprehensive study was carried out to understand the pattern in intraspecific variation, but found no specific adaptations to environment (Kaerger et al. 2015).

*R. arrhizus* is found all over the world, colonizing a wide range of ecological habitats. Most strains of *R. arrhizus* are saprotrophic inhabitants of soil, dung, organic matter, and plant debris, while some isolates parasitize plants and infect animals and humans, causing mucormycosis, especially those co-infected with COVID-19 (Zheng et al. 2007; Kwon et al. 2011; Cheng et al. 2017; Ju et al. 2020; Tabarsi et al. 2021). The species plays an important role in industrial bio-transformations, such as beverage and food processing through fermentation (Jin et al. 2003; Liu et al. 2017; Dobrev et al. 2018). Meanwhile, *R. arrhizus* has a strong ability to uptake a wide range of substrates, including rice meal, soybean meal, glucose, and corn pulp, as feedstock to yield many productions that were widely used in food, therapeutic compounds and other biotechnological industries (Liu et al. 2017; Yao et al. 2018; López-Fernández et al. 2021; Bai et al. 2022; Corzo-León et al. 2023).

High-throughput phenotyping is a cornerstone of system biology, allowing simultaneous measurements of numerous taxa and characters (Cuevas and Edwards 2018). The Biolog automatic microbial analysis system, one of the high-throughput phenotypic assays, is able to identify organisms rapidly, effectively and accurately (Yao et al. 2006). This system, especially the FF MicroPlate designed particularly for filamentous fungi, is used to test the utilization of as many as 95 kinds of carbon/nitrogen sources at the same time (Tang et al. 2015; Schwendner and Schuerger 2018; Kabtani et al. 2024). For instance, the ability to metabolize most carbon/nitrogen sources was found to be lost at a pressure of 0.7 kPa in *Sarrella liquefaciens* (Schwendner and Schuerger 2018). Biolog system was also used to estimate resistance/sensitivity and metabolic activities against fungicides in filamentous fungi, such as *R. arrhizus*, *Botrytis cinerea*, and *Fusarium* spp. (Frąc et al. 2016; Wang et al. 2016, 2018). Previous study showed that Biolog FF plates facilitated optimizing media for *R. arrhizus* and *Rhizopus microsporus* (Kordowska-Wiater et al. 2012). Closely related fungal strains were able to be discriminated using Biolog FF plates, e.g., those in *Aspergillus* spp., *Coprinus comatus*, *Ganoderma lucidum*, *Oidiodendron fimicola*, and *Petriella setifera* (Rice and Currah 2005; Singh 2009; Pawlik et al. 2015a, 2015b; Rola et al. 2015; Pertile et al. 2018).

In this study, we applied the Biolog FF MicroPlate to a diverse culture collection of the filamentous fungus *R. arrhizus*, assessing its diversity in carbon/nitrogen source assimilation capacity, screening for strains of potential use for industrial fermentations, as well as estimating optimum substrates for its growth and sporulation.

## Materials and methods

### Strain collections

A total of 69 strains of *R. arrhizus* were collected from five continents covering 21 countries (Table 1). They were isolated from clinical, domesticated, and natural samples, such as skin, insects, flowers, koji, cakes, wrappers, air, and soil. Cultures were preserved at – 80 °C in 15% glycerol at China General Microbiological Culture Collection Center (CGMCC, China), Westerdijk Fungal Biodiversity Institute (CBS, the Netherlands), Shandong Normal University (XY, China), and University of Michigan (UM, USA). And more information of 69 strains of *R. arrhizus* were listed in Table 1.

**Table 1:** Information of involved strains of *Rhizopus arrhizus*

Strains	Deposited no	Varieties	Origin	Geography	Samples	AWCD	SR	PCA	Sporulation
XY00077	CGMCC 3.15794	*Delemar*	Clinical	China	Skin scabs	0.46	332		Yes
XY00406	CBS 387.34	*Arrhizus*	Domesticated	Japan	Koji	0.45	324		Yes
XY00409	CBS 330.53	*Tonkinensis*	Wild	Japan	Soil	0.45	347		Yes
XY00419	CBS 389.34	*Delemar*	Wild	Japan	Ragi	0.21	239		Yes
XY00424	CBS 328.47	*Arrhizus*	Domesticated	Japan	Koji	0.35	321		Yes
XY00438	CBS 258.28	*Tonkinensis*	Domesticated	China	Chinese yeast	0.61	352		Yes
XY00457	CBS 110.17	*Arrhizus*	Wild	Portugal	Corn flour	0.53	357		Yes
XY00495	CBS 279.38	*Delemar*	Domesticated	India	Distillery yeast	0.62	450		Yes
XY00507	CBS 128.08	*Arrhizus*	Domesticated	China	Chinese yeast	0.46	394		No
XY01735	CGMCC 3.9500	*Delemar*	Wild	China	Soil	0.50	307		Yes
XY01736	CGMCC 3.9501	*Arrhizus*	Wild	China	Flour	0.59	389		No
XY01737	CGMCC 3.9502	*Delemar*	Wild	China	Flower	0.54	392		Yes
XY01738	CGMCC 3.9503	*Delemar*	Clinical	China	Cake	0.57	322		Yes
XY01745	CGMCC 3.1136	*Delemar*	Wild	China	Air	0.50	328		Yes
XY01857	CGMCC 3.9478	*Arrhizus*	Wild	China	Flower	0.13	178		Yes
XY01864	CGMCC 3.9516	*Arrhizus*	Wild	China	Flower	0.48	294		Yes
XY01865	CGMCC 3.9480	*Delemar*	Wild	China	Sweet wrapping	0.27	276		Yes
XY01874	CGMCC 3.9518	*Arrhizus*	Wild	China	Grass	0.66	420		Yes
XY01875	CGMCC 3.9483	*Delemar*	Wild	China	Wrapping paper	0.60	378		Yes
XY01876	CGMCC 3.9519	*Arrhizus*	Wild	China	Soil	0.60	318		Yes
XY01880	CGMCC 3.9484	*Delemar*	Wild	China	Soil	0.57	389		Yes
XY01919	CGMCC 3.9492	*Arrhizus*	Wild	China	Plant	0.39	271		Yes
XY01920	CGMCC 3.9493	*Delemar*	Clinical	China	Lesion	0.36	291		Yes
XY01921	CGMCC 3.9531	*Arrhizus*	Clinical	China	Eye socket	0.44	285		Yes
XY01957	CGMCC 3.9510	*Arrhizus*	Domesticated	China	Distillery yeast	0.17	202		Yes
XY02053	CGMCC 3.15792	*Tonkinensis*	Wild	China	Sweet wrapping	0.47	281		Yes
XY02064	CGMCC 3.15793	*Tonkinensis*	Wild	China	Soil	0.45	247		Yes
XY02120	CGMCC 3.9533	*Arrhizus*	Wild	China	Shell	0.44	281		Yes
XY02128	CGMCC 3.9534	*Tonkinensis*	Wild	China	Dung	0.55	286		Yes
XY03778	UM 1059	*Delemar*	Domesticated	Tanzania	*Vigna unguiculata*	0.32	280		Yes
XY03779	UM 1060	*Arrhizus*	Wild	United Kingdom	Lake mud	0.49	306		Yes
XY03782	UM 738	*Arrhizus*	Wild	Indonesia	Ragi	0.31	276		Yes
XY03785	UM 778	*Arrhizus*	n.a	USA	n.a	0.39	220		No
XY03786	UM 780	*Arrhizus*	Wild	Cyprus	*Vicia faba* seedling	0.59	287		Yes
XY03787	UM 781	*Tonkinensis*	Wild	Egypt	*Allium*	-0.18	55	Outlier	Yes
XY03788	UM 782	*Delemar*	Wild	India	*Gossypium*	0.58	316		Yes
XY03789	UM 783	*Arrhizus*	Wild	Malaysia	Honey dew	0.59	380		Yes
XY03790	UM 786	*Arrhizus*	Wild	Yemen	*Gossypium* root	0.51	297		Yes
XY03792	UM 787	*Delemar*	Domesticated	Malaysia	Soy sauce	0.56	389		Yes
XY03793	UM 788	*Delemar*	Wild	Malaysia	*Pinus caribaea*	0.45	287		Yes
XY03794	UM 789	*Delemar*	Wild	India	Cotton lint	0.52	376		No
XY03795	UM 791	*Tonkinensis*	Wild	Egypt	*Arachis hypogaea*	0.47	293		Yes
XY03796	UM 792	*Tonkinensis*	Wild	Jordan	*Allium*	0.55	294		Yes
XY03797	UM 793	*Arrhizus*	Wild	Papua New Guinea	Radio set	0.67	349		Yes
XY03798	UM 794	*Delemar*	Wild	Venezuela	*Carica papaya* fruit	0.56	312		Yes
XY03799	UM 795	*Arrhizus*	Wild	Uganda	Mouldy bran	0.60	305		Yes
XY03800	UM 925	*Delemar*	Wild	The Philippines	Soil	0.53	274		Yes
XY03801	UM 926	*Delemar*	Wild	South Africa	Soil	0.63	393		No
XY03802	UM 927	*Delemar*	Wild	The Philippines	Soil	0.37	316	Outlier	Yes
XY03803	UM 928	*Delemar*	Wild	The Philippines	Soil	0.65	360		Yes
XY03804	UM 929	*Delemar*	Wild	Sudan	Dung	0.37	287		Yes
XY03805	UM 930	*Delemar*	Wild	Indonesia	Ragi-tempeh	1.07	470		Yes
XY03806	UM 931	*Tonkinensis*	Clinical	USA	Clinical	0.95	499	Outlier	Yes
XY03808	UM 933	*Tonkinensis*	Wild	USA	Sweet potato	0.90	478		Yes
XY03809	UM 934	*Delemar*	Wild	Pakistan	Dung	0.82	492		Yes
XY03810	UM 935	*Delemar*	Clinical	USA	Clinical	0.53	391		Yes
XY03813	UM 938	*Tonkinensis*	Wild	USA	Soil	0.66	357		Yes
XY03815	UM 940	*Delemar*	Wild	Uganda	Peanuts	0.31	251		Yes
XY03816	UM 941	*Delemar*	Wild	The Philippines	Soil	0.36	248		Yes
XY03819	UM 944	*Arrhizus*	Wild	India	Insect	0.54	337		Yes
XY03820	UM 945	*Arrhizus*	Wild	Iran	Onion	0.56	335		Yes
XY03821	UM 946	*Delemar*	Wild	Indonesia	Leaf	0.42	263		Yes
XY03822	UM 947	*Delemar*	Wild	South Africa	Soil	0.64	389		No
XY03824	UM 950	*Delemar*	Wild	The Philippines	Soil	0.31	242		Yes
XY03825	UM 951	*Delemar*	Wild	Indonesia	Soil	0.39	271		Yes
XY03826	UM 952	*Delemar*	Wild	Indonesia	Soil	0.36	247		Yes
XY03827	UM 953	*Delemar*	Wild	Indonesia	Soil	1.29	519		Yes
XY03829	UM 955	*Delemar*	Wild	Pakistan	Dung	0.55	349		No
XY03830	UM 956	*Delemar*	Wild	Indonesia	Food	0.66	367		Yes

*AWCD* the average well color development data on the 4th day, *SR* the total substrate richness values for all the incubation time of 7 days. *Sporulation* the strain can (yes) or cannot (no) produce sporangiospores after a standing incubation at 30 °C for 20 d in flasks with liquid medium SML. *n.a* not available. AWCD ≥ 0.82, SR ≥ 420, outliers in PCA and non-sporulation are shaded

### Morphological and phylogenetical analyses

The 69 strains collected were classified into three varieties: *Rhizopus* var. *arrhizus*, var. *tonkinensis*, and var. *delemar* (Table 1), based on a combination of morphology and DNA sequences targeting the ITS and IGS rDNA (Dolatabadi et al. 2014; Liu et al. 2008; Zheng et al. 2007).

*R. arrhizus* strains were cultured on potato dextrose agar (PDA: potato 200 g/L, glucose 20 g/L, agar 20 g/L) plates, and incubated at 30 °C. The morphology of *R. arrhizus* was observed under an optical microscope (Zeiss IMAGER A2-M2, Oberkohen, Germany) according to the description by Zheng et al. (2007).

Genomic DNA was extracted from cultures using the Biospin Fungus Genomic DNA Extraction Kit (Bioer Technology Company Limited, Beijing, China), following the provided instructions. The primer pairs NS5M (5′-AAC TTA AAG GAA TTG ACG GAA G-3′)/LR5M (5′-TCC TGA GGG AAA CTT CG-3′) and NR5688F (5′-GAG TAG CCT TTG TTG CTA CG-3′)/RGR-1 (5′-TTC TAG GTG ATG GAC GGC-3′) were used to amplify ITS and IGS rDNA, respectively (Liu et al. 2008; White et al. 1990). The polymerase chain reaction (PCR) mixture (25 μL) included 1 μL of fungal DNA template (10 ng/μL), 1 μL of forward and reverse primers each (5 μM), 12.5 μL of Ex Taq MasterMix (2×), and 9.5 μL of ddH_2_O. The polymerase chain reaction (PCR) program was performed as follows: an initial denaturation at 94 °C for 5 min, followed by 33 cycles of 94 °C for 30 s, 60 °C for 45 s, and 72 °C for 45 s, and a final extension at 72 °C for 10 min. PCR products were verified by 1% agarose gel electrophoresis and sequenced at Shanghai Meiji Bio-pharmaceutical Company (Shanghai, China) using primers ITS5 (5′-GGA AGT AAA AGT CGT AAC AAG G-3′)/ITS4 (5′-TCC TCC GCT TAT TGA TAT GC-3′) for internal transcribed spacer (ITS) rDNA and NR5688F/RGR-1 for 28S-18S ribosomal RNA intergenic spacer (IGS) rDNA, respectively (Liu et al. 2008; White et al. 1990). Sequence reads were manually checked and assembled with Geneious 8.1.9 (https://www.geneious.com/, Kearse et al. 2012), and a Maximum Likelihood (ML) phylogenetic tree with 1000 bootstrap replicates based on ITS and IGS rDNA was reconstructed using the GTRGAMMA model through the raxmlGUI 2.0 (Edler et al. 2021) implemented in Geneious 8.1.9 (Kearse et al. 2012). Finally, the phylogenetic trees (Supplemental Figs. S1 and S2) were viewed in iTOL 6 (https://itol.embl.de, Letunic and Bork 2024).

### Strain sporulation

Strains were revived on malt extract agar (MEA: malt extract 20 g/L, peptone 1 g/L, glucose 20 g/L, agar 20 g/L, pH 7.0), at 30 °C for a duration of 2 days. Aerial hyphae were then delicately scraped off using inverted 200 μL pipette tips, and 5 mm diameter inoculates were obtained by punching the inverted tip into the medium. Single inoculate was cultivated at 30 °C for 3 days in 250 mL flasks containing 20 mL of synthetic *Mucor* liquid (SML: glucose 20 g/L, asparagine 2 g/L, K_2_HPO_4_ 0.5 g/L, MgSO_4_⋅7H_2_O 0.25 g/L, vitamin B_1_ 0.5 mg/L). For strains showing little or no sporangiospore productions, cultivation extended up to 20 days. Notably, five *R. arrhizus* strains (XY00507, XY01736, XY03794, XY03822, and XY03829, Table 1) were further examined due to their lack of sporulation in the SML medium.

In cases where strains did not produce sporangiospores, inoculation onto MEA solid media was employed to collect sporangiospores for subsequent suspension in filamentous fungus inoculating fluid (FF-IF) medium. Under a stereomicroscope (Leica, MDG33, Singapore), the Biolog FF MicroPlate was meticulously examined. Each well was individually photographed after 7 days of growth to ensure sufficient sporulation. Sporangiospores from each well were eluted three times with 200 μL of sterile water and transferred into 1 mL Eppendorf (EP) tubes. Hemocytometer counts and microscopic observations (Zeiss, Axio Imager A2, Oberkohen, Germany) were employed to determine spore concentrations. Strains with spore concentrations of 0, 1.50E5^−5.25E5^, 8.75E5^−2.00E6^, and 2.50E6^−1.14E7^ sporangiospores/mL were categorized as non-promoted, poorly promoted, moderately promoted, and heavily promoted strains, respectively. Substrate promotion frequency (PF) and high promotion frequency (HPF) denoted the proportions of promoted and heavily promoted strains among all five tested strains, respectively.

### High-throughput screening carbon/nitrogen sources

In this study, we used well absorbance values, biological activities, strain substrate richness, and substrate utilization frequency to estimate carbon/nitrogen assimilations of *R. arrhizus* strains through FF MicroPlates containing 95 different carbon and nitrogen sources.

Strains were revived on solid media MEA at 30 °C for 4 days or until sporangia were abundantly produced. Sporangiospores were swabbed off the agar media using a sterile cotton swab pre-macerated with filamentous fungus inoculating fluid (FF-IF Biolog catalog #72,106, 0.25% Phytagel and 0.03% Tween 40 in DI water, Biolog Inc., Hayward, CA, USA), avoiding carryover nutrients from the agar medium, and then they were suspended into a 10 mL tube containing 5 mL of FF-IF. Suspensions were homogenized by adjusting to an optical density (OD) value of 0.15 at a wavelength of 590 nm with a 752 UV Visible Spectrophotometer (L1611031, Jinghua Technology Instrument Company, Shanghai, China). Then, 100 μL of homogenized suspensions were pipetted into each well of the FF MicroPlate (Table 1, Biolog catalog #1006, Biolog Inc., Hayward, USA) and then incubated in the absence of light at 30 °C for 7 days. Experiments were carried out in three technical repeats for each strain. OD values of the FF MicroPlates were read in triplicate daily using a Biolog GEN III MicroStation Reader (Cat. No. S/N E 11388, Biolog Inc., Hayward, USA) at 490 and 750 nm for determining absorbance and turbidity, respectively. All OD values were controlled by zero carbon/nitrogen (A1: Water in Supplemental Table S1) and calibrated by initial values.

The biological activity of the strains was quantified through an overall source utilization metric, which encompassed the calculation of the average well color development (AWCD, represented the average well color development data on the 4th day). The AWCD index was adjusted by subtracting values obtained from the blank (water) and calculated using the formula AWCD = [Σ(Ci − R)]/95, where 95 represented the number of wells or substrates, Ci denoted the optical density of substrate measured at 490 nm, and R represented the optical density of the control zero-carbon well (Garland and Mills 1991). To facilitate comparisons between different groups, a one-way MANOVA analysis focusing on AWCD was conducted for three varieties (var. *arrhizus*, var. *delemar*, and var. *tonkinensis*) and three origins (clinical, domesticated, and natural) of strains (Table 1).

When the difference between a well’s absorbance and its control value (C–R) exceeded 0.25 or 1.50, the corresponding carbon/nitrogen source was classified as an efficient or highly efficient substrate, respectively (Zou et al. 2016). The substrate utilization frequency (UF) and substrate high utilization frequency (HUF) were defined as the proportions of strains utilizing efficient and highly efficient substrates, respectively. These proportions were calculated as N/69, where 69 represented the total number of tested strains, and N denoted the count of strains utilizing efficient or highly efficient substrates. For comparative analysis across different groups, a one-way MANOVA focusing on UF was conducted for two types (carbon and nitrogen) or six substrate categories (alcohols, amines, amino acids, carbohydrates, carboxylic acids/derivatives, and glycosides, as per the classification from the DrugBank database (https://www.drugbank.ca/, Wishart et al. 2018).

### Data analyses

The principal component analysis (PCA) and visualization of substrate utilization data were conducted using the web tool ClustVis (https://biit.cs.ut.ee/clustvis/, Metsalu and Vilo 2015). As the AWCD demonstrated a gradual increase followed by a stabilization from the 4th day onwards, PCA analyses and fingerprint mapping were based on absorbance values recorded at the 4th day. These values, controlled by zero carbon/nitrogen conditions and calibrated with the initial measurement, served as the foundation for the analyses.

In the PCA analyses, strains were considered as annotations, rows were scaled with unit variance, and principal components were calculated through the default singular value decomposition (SVD) method with imputation implemented in ClustVis website (https://bit.cs.ut.ee/clustvis/, Metsalu and Vilo 2015). Prediction ellipses were drawn at a 95% confidence level, and the X- and Y-axes were constrained to share the same scale. For heatmap creation, rows and columns were both clustered using correlation distance with single-linkage criterium, two-color gradient, and unit variance scaling.

Venn diagrams were generated using the OriginPro 2020 (https://www.originlab.com/, Seifert 2014). The sporulation promotion frequency (PF), high promotion frequency (HPF), growth utilization frequency (UF), and high utilization frequency (HUF) data of substrates were imported into the software as sheets, and the range was specified based on respective values. The process involved selecting the “Venn Diagram” app implemented within OriginPro to create the graphical representation. Additionally, adjustments to the color and labeling of each set in the Venn diagram were made to align with the specific requirements of the data.

In addition, the statistical analysis was carried out using IBM SPSS Statistics for Windows, version 19.0 (IBM Corp., Armonk, N.Y., USA).

## Results

### Carbon and nitrogen assimilation abilities of *Rhizopus arrhizus*

**Fig. 1:**
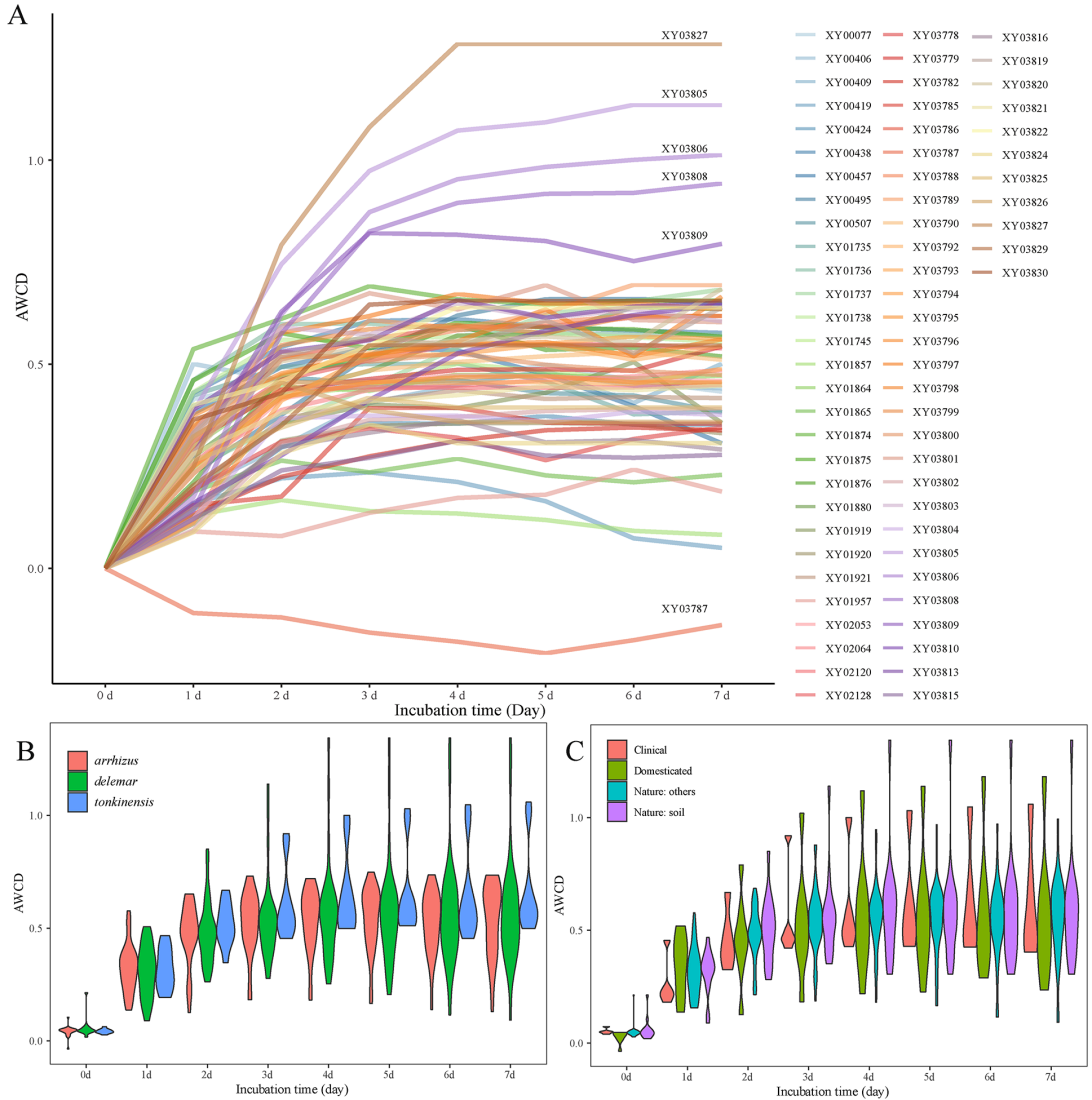
Average well color development (AWCD, y-axis) against incubation time (x-axis). **A** The trend for 69 strains of *R. arrhizus* showing their carbon/nitrogen assimilation abilities while being incubated with Biolog FF MicroPlate for 7 days. AWCD values are means (standard deviations not shown) from three independent technical repeats each being read in triplicates. **B** The carbon/nitrogen assimilation trend for three varieties (var. *arrhizus*, var. *tonkinensis*, and var. *delemar*) of *R. arrhizus*. **C** The carbon/nitrogen assimilation trend for three origins (clinical, domesticated, and natural) of *R. arrhizus*

The absorbance values for the 69 *R. arrhizus* strains across 96 wells were carefully monitored and calibrated over a 7-day period. The average well color development (AWCD) exhibited a swift increase during the exponential phase from the beginning to the 4th day, and a nearly stable trend in the stationary phase from the 4th to the 7th day (Fig. 1A).

The *R. arrhizus* strains displayed diverse biological activities in utilizing carbon/nitrogen sources. Analyzing the data on the 4th day (Fig. 1A and Table 1), which corresponds to the onset of the stationary phase, the strains were classified into three categories: (1) five strains with higher AWCD values (0.82–1.29), specifically XY03827, XY03805, XY03806, XY03808, and XY03809, (2) 63 strains with moderate AWCD values (0.13–0.67), representing the majority of the species, (3) only one strain, XY03787, with the lowest AWCD value (− 0.18).

MANOVA analyses revealed no significant differences (*P* > 0.05) among varieties, origins, and substrate richness of *R. arrhizus* strains (Figs. 1B, C, S3; Table 1). Seven strains possessed a higher number of 7-day-efficient substrates (SR = 420–519), including XY03827, XY03806, XY03809, XY03808, XY03805, XY00495, and XY01874. The strain XY03787 was the lowest (SR = 55) and the other 61 strains were moderate (SR = 178–394).

**Fig. 2:**
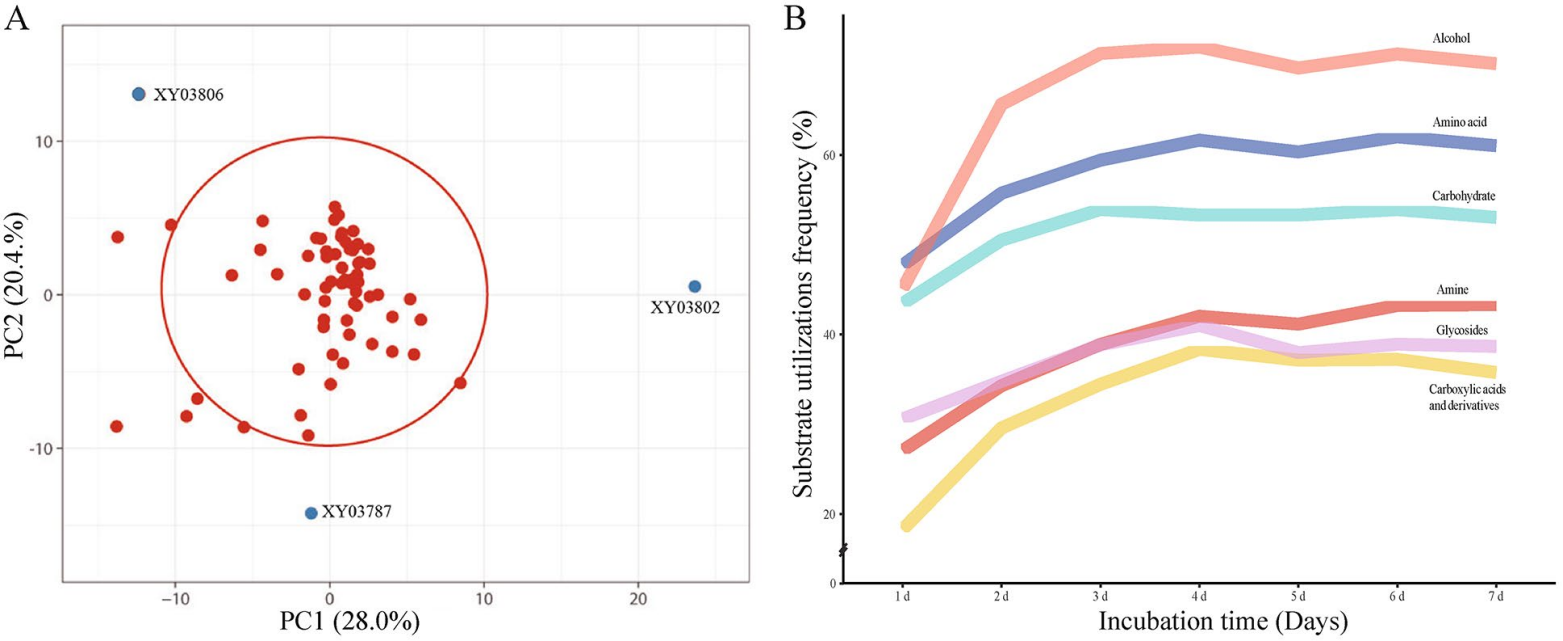
Principal component analysis (PCA) and utilization frequency (UF) of *Rhizopus arrhizus*. **A** PCA of the 4th day’s absorbance values for 69 strains of *Rhizopus arrhizus* while being incubated with Biolog FF MicroPlate. X and Y axes show PC1 and PC2 that explain 28.0 and 20.4% of the total variance, respectively. Three outliers are labeled as blue points and strain’s codes, while other strains are only labeled as red points and encompassed with a 95% prediction ellipse. **B** The curve of utilization frequency (UF, Y-axis) of six categories of substrates by *Rhizopus arrhizus* during an incubation period of 7 days (X-axis). The highest efficient alcohols are significantly different from four categories (*P* = 2.28E^−4^ for carbohydrates, 4.81E^−5^ for amines, 9.58E^−7^ for glycosides, 6.15E^−11^ for carboxylic acids and derivates), except the amino acids (*P* = 0.14)

The principal component analysis (PCA, Fig. 2A) of 69 strains of *R. arrhizus* revealed that the primary sources of variability were captured by the two components (PC1 and PC2) based on the controlled and calibrated absorbance values on the 4th day. The majority of strains formed a cohesive cluster, showcasing similar patterns in carbon/nitrogen source utilization. While three strains, namely XY03787, XY03802, and XY03806, were outlier strains, indicating distinctive patterns. In summary, 13 strains were unique in PCA, heatmap, AWCD, and SR analyses, which may imply a niche differentiation within *R. arrhizus* (Supplemental Table S2).

### Utilization of carbon/nitrogen sources

When considering the 95 substrates individually, their 7-day average utilization frequencies (UFs) exhibited a continuous distribution ranging from 0.41 to 97.13% (Supplemental Table S1). High utilization frequencies (HUFs) were categorized into two groups (0–39.66% and 54.47–84.99%, Supplemental Table S1). Among the 95 substrates, seven demonstrated UFs exceeding 95%, namely *N*-acetyl-d-galactosamine (A03), sedoheptulosan (E03), d-tagatose (E08), l-alanamine (G07), l-alanyl-glycine (G09), glycyl-l-glutamic acid (H01), and l-phenylalanine (H03). Additionally, two substrates, *N*-acetyl-d-galactosamine (A03) and l-phenylalanine (H03), displayed HUFs exceeding 75%. MANOVA analyses did not reveal significant differences between the carbon and nitrogen types of substrates (*P* > 0.05). When considering six categories of substrates (Supplemental Table S1), significant differences in UFs were observed across most categories (*P* < 0.01, Fig. 2B). Alcohols emerged as the most highly utilized category, followed sequentially by amino acids, carbohydrates, amines, glycosides, and carboxylic acids/derivatives.

### Carbon/nitrogen sources promoting sporulation

**Fig. 3:**
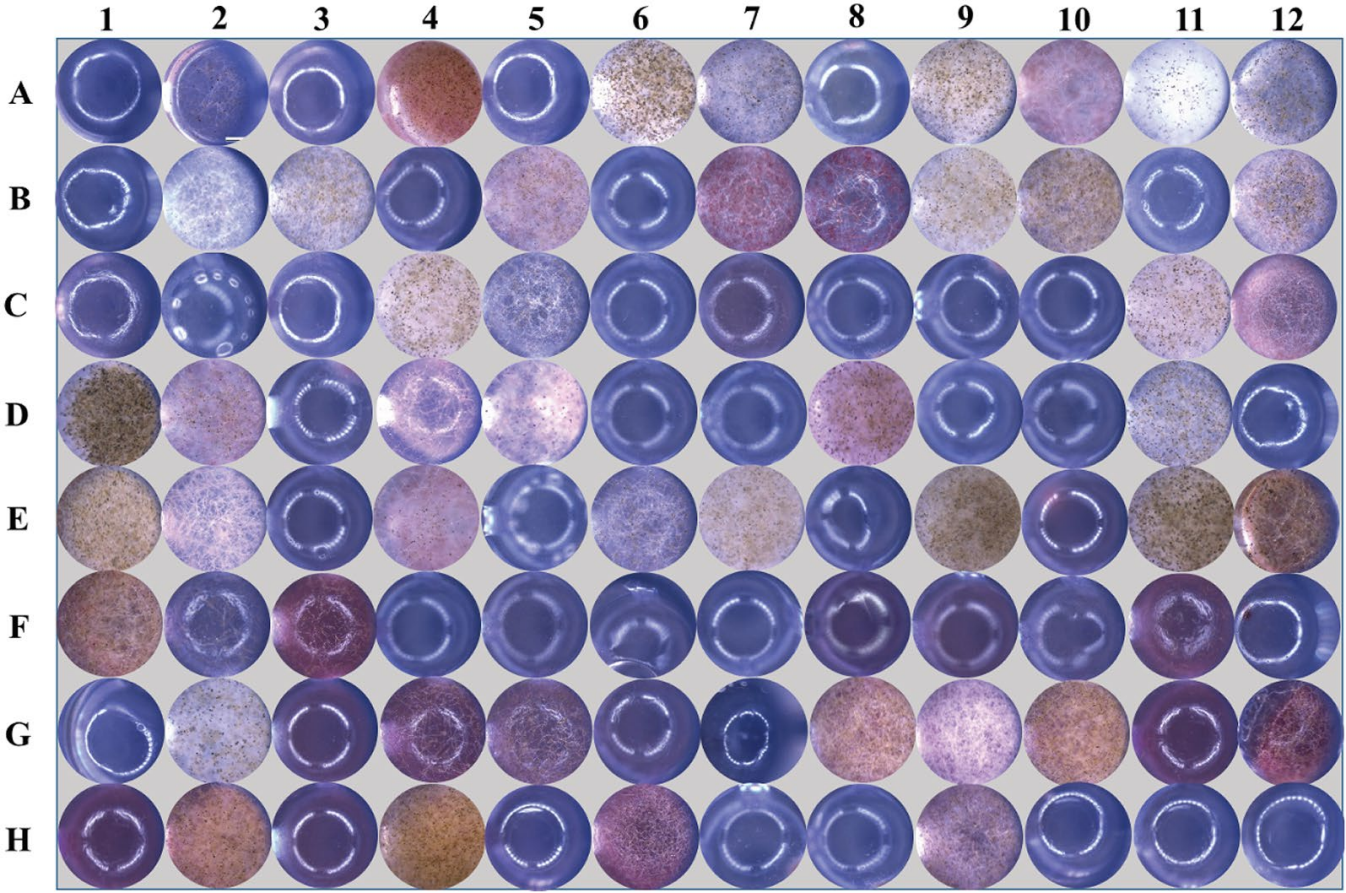
The macroscopic sporulation of XY03794 inoculated with the discrete carbon/nitrogen substrates fixed on the Biolog FF MicroPlate with 8 rows (**A**–**H**) and 12 columns (1–12). The detail of the carbon/nitrogen substrates refers to Supplemental Table S1

Sporangiospore production was not observed in seven out of the 69 strains, even after an extended incubation period of 20 days (Table 1). Among these seven strains, five (XY00507, XY01736, XY03794, XY03822, and XY03829) were selected for additional investigations into the influence of various substrates on sporulation (Table 1). Microscopic examination of the culture revealed that eight substrates consistently promoted sporulation in all strains (PF = 100%), including *N*-acetyl-d-glucosamine (A04), chunfushoucao (A06), l-arabinose (A09), glycerin (C04), maltose (C11), d-sorbitol (E04), xylitol (E11), and sebacic acid (G02). Approximately 40 substrates stimulated sporulation in some strains, with 10 of them highly promoting sporulation in over half of the strains (HPF = 60%). The remaining 47 substrates showed no stimulation of sporulation (Supplemental Table S1). The variation in substrate effectiveness in facilitating sporulation is shown using the example of the strain XY03794 (Fig. 3).

### Substrate preference of *Rhizopus arrhizus*

**Fig. 4:**
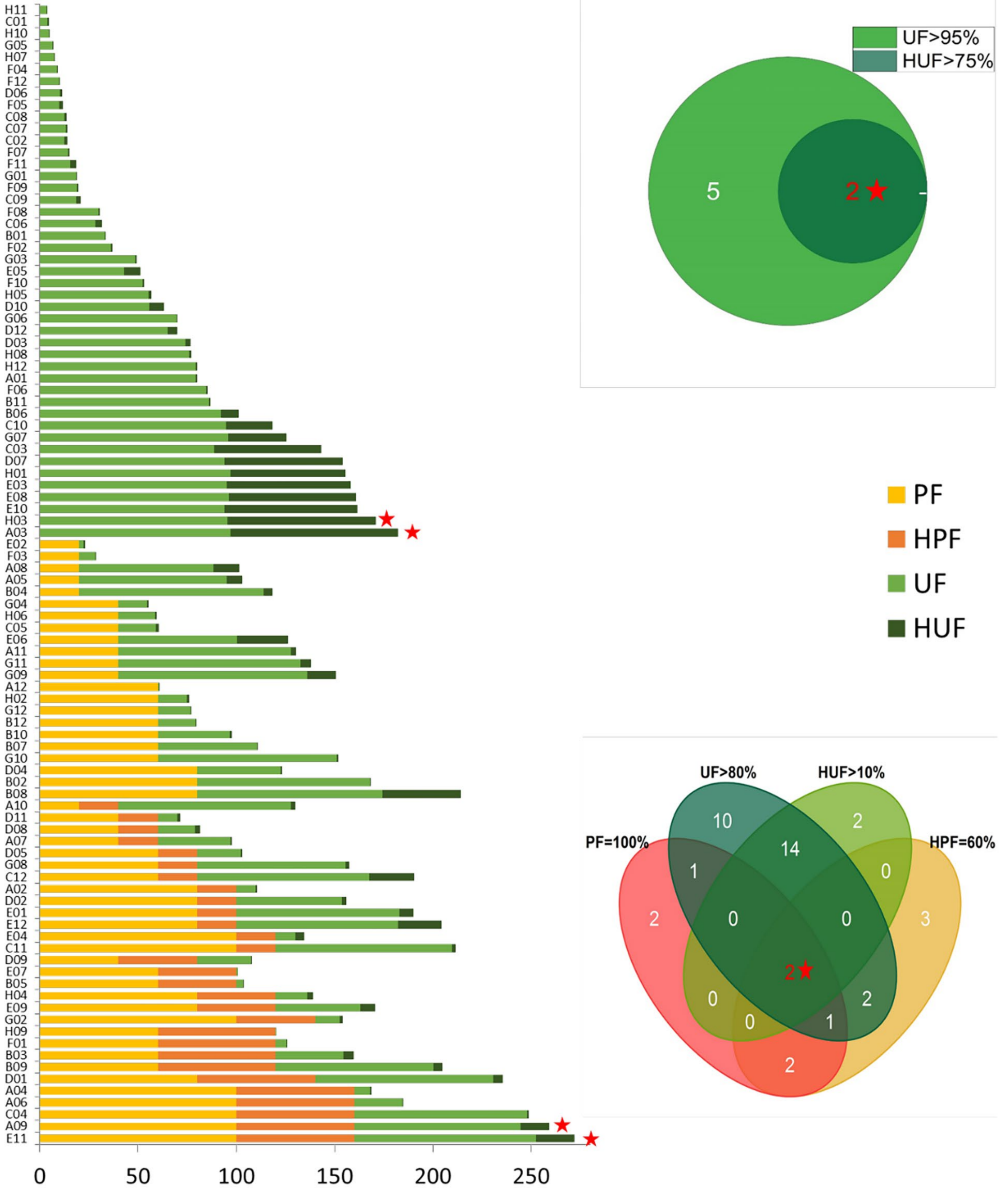
The capability of 95 carbon/nitrogen substrates for promoting growth and sporulation. The bar chart on the left shows the accumulative percentage of sporulation promotion frequency (PF), high promotion frequency (HPF), growth utilization frequency (UF) and high utilization frequency (HUF). The Venn diagram on the upper right indicates the relationship between the strains with a UF > 95% or HUF > 75%, and that on the lower right displays those with a UF > 80%, HUF > 10%, PF = 100% or HPF = 60%. The red stars highlight the best substrates also revealed in relevant Venn diagrams, two for growth (A03: *N*-acetyl-d-galactosamine and H03: l-phenylalanine) and two for sporulation (A09: l-arabinose and E11: Xylitol). The detail of carbon/nitrogen refers to Supplemental Table S1

*R. arrhizus* has a preference for specific substrates, as demonstrated by variations in the average utilization frequency (UF) across six type substrates. A one-way MANOVA analysis revealed a significant difference (*P* < 0.01), with alcohols being the most preferred type (Fig. 2B). Notably, certain high-utilization substrates, particularly *N*-acetyl-d-galactosamine (A03) and l-phenylalanine (H03), with a UF > 95% and HUF > 75%, were identified as favorable for the growth of *R. arrhizus* (Fig. 4 and Supplemental Table S1). This substrate preference is instructive for the formulation and optimization of selective media (Pertile et al. 2018).

Beyond influencing the growth of *R. arrhizus*, various substrates were found to promote sporulation. When considering non-sporulation strains, only 50 out of the 95 substrates (53%) demonstrated the ability to induce sporulation (Fig. 4 and Supplemental Table S1). Among the 95 substrates, l-arabinose (A09) and xylitol (E11) emerged as the most efficient substrates, meeting a combined criterion of UF > 80%, HUF > 10%, PF = 100%, and HPF = 60%. These substrates hold potential for rejuvenating degenerated strains that have lost the ability to sporulate (Supplemental Fig. S4).

### Interspecific diversity analyses

**Fig. 5:**
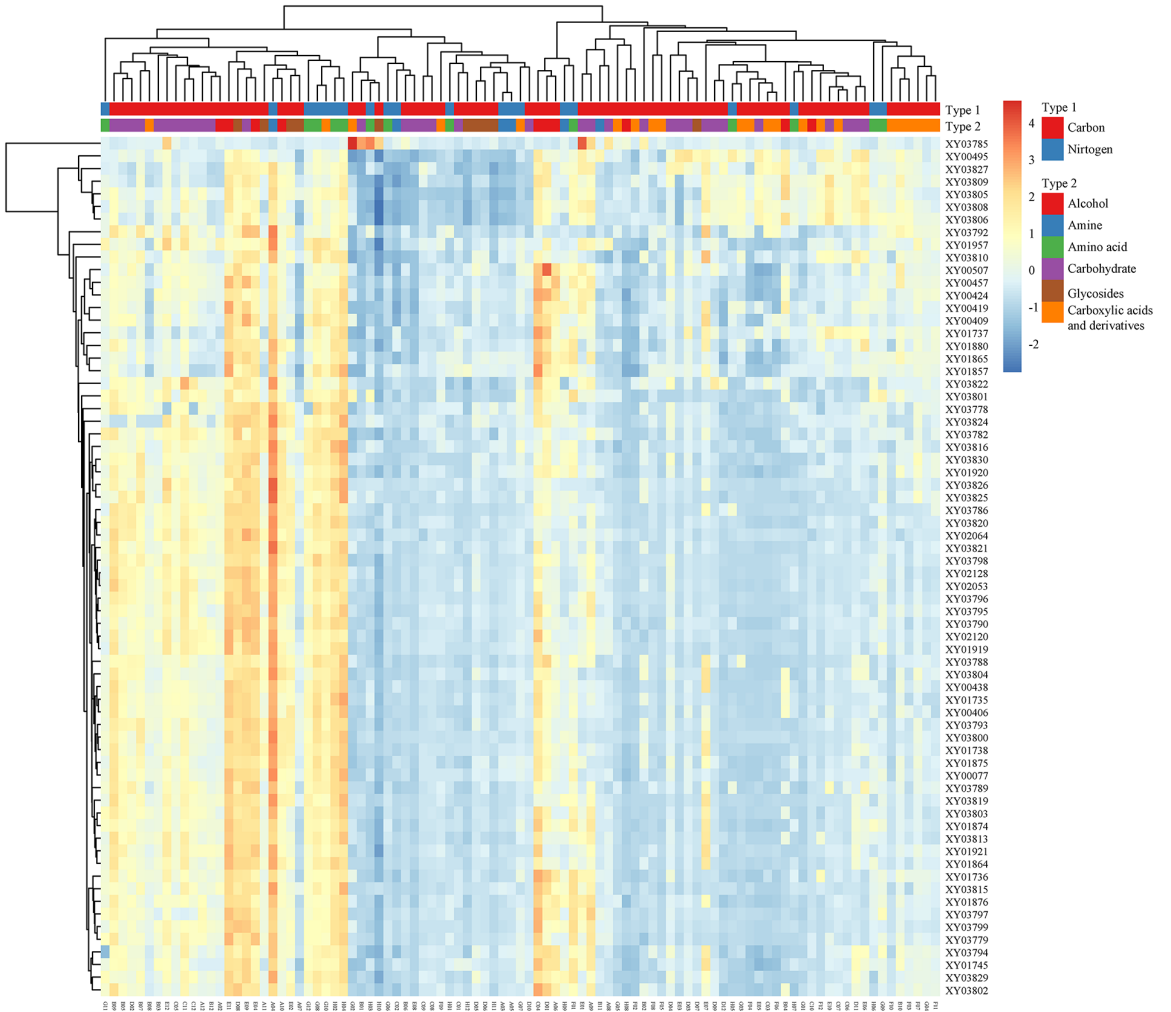
The clustered heatmap of the absorbance values at the 4th day for 69 strains of *Rhizopus arrhizus* (row) against 95 substrates (column) showing inter-strain and inter-substrate variations. The trees on the left and top illustrate clustering of the strains and substrates, respectively. Annotations about the carbon/nitrogen substrates are labeled on the top and their detail refers to Supplemental Table S1

Distinct variations in the fingerprints of carbon/nitrogen source metabolisms were evident in *R. arrhizus* (Fig. 5). While there were no clear boundaries separating carbon sources from nitrogen sources, the 95 carbon/nitrogen sources could be roughly categorized into two groups, as indicated by the two clades on the substrate phylogram (Fig. 5), which inconsistent with the morphology and molecular evidence (Zheng et al. 2007; Liu et al. 2008). Approximately 59% (56/95) of these sources exhibited low utilization, depicted in bluish tones and grouped on the left side of Fig. 5, whereas 41% (39/95) were highly utilized, represented in reddish tones and positioned on the right.

In the phylogenetic trees (Supplemental Figs. S1 and S2), four major clades were identified. Apart from the primary clade containing the majority of strains, three sub-clades highlighted nine strains with specific substrate assimilation patterns. These strains were XY03801, XY03806, XY03799, XY00495, XY03805, XY00077, XY03827, XY03813, and XY03809.

## Discussion

Many microorganisms, such as *Mucor*, *Rhizopus*, *Saccharomyces*, and *Streptococcus* species, play an important role in the fermentation process to yield a wide range of bio-productions (Blandino et al. 2003; Vagelas et al. 2011; Sharma et al. 2020; Mokoena 2017; Ashaolu et al. 2023). Among these species, *R. arrhizus* usually participates in traditional food fermentation, including tempeh, peka, baijiu, ragi, loog-pang, rice wine, and rice vinegar (Kitpreechavanich et al. 2008; Abd Razak et al. 2017; Mokoena 2017; Corzo-León et al. 2023). During the fermentation, it is well-known for producing lactic acid, fumaric acid, malic acid, ethanol, cellulase, amylase, and lipase (Garlapati and Banerjee 2010; Chotisubha-anandha et al. 2011; Kaur et al. 2015; Kupski et al. 2015; Yao et al. 2018; Bai et al. 2022; Corzo-León et al. 2023,). Previous studies suggest that *R. arrhizus* can utilize a large number of substrates for fermentation (Liu et al. 2016; Londoño-Hernández et al. 2017; Dobrev et al. 2018; López-Fernández et al. 2021; Bai et al. 2022), or yield desired metabolites by optimizing cultivation conditions (Lhomme and Roux 1991; Chotisubha-anandha et al. 2011; Liu et al. 2017). In this study, our results propose that eight carbon and nitrogen substrates significantly promote the sporulation of *R. arrhizus*, which will be beneficial for application in fermentation.

Fungal degeneration entails the spontaneous loss or decline in production capacity, resulting in significant economic losses. This phenomenon poses a threat to some of the widely utilized fungal genera in the biotechnical industry, such as *Aspergillus*, *Penicillium*, and *Trichoderma* (Adrio and Demain 2003; Douma et al. 2011; Song et al. 2022; Danner et al. 2023). Similar with these fungi, as the number of passages increases and artificial domestication of fermentation environment is prolonged, certain *R. arrhizus* strains may progressively undergo degradation, which is primarily characterized by a sluggish expansion of mycelia, a reduction in sporangiospore quantities, and even a complete absence of sporangiospore production. The degradation of *R. arrhizus* strains will also hinder the application in food and industry. Previous studies suggest that strains can be reduced through various pathways, such as preservation methods, strains selection, and bioprocess design (Al-Bedak et al. 2019; Meyer et al. 2021; Danner et al. 2023). Our study shows that eight substrates can significantly promote the reproduction of sporangiospores in some strains of *R. arrhizus*, which cannot sporulate even after an extended incubation period of 20 days with the synthetic *Mucor* liquid (SML).

In addition, this study adopts a quick and easy way to obtain the metabolic profile of various carbon/nitrogen sources, which was illustrated by Yao et al. (2018). Single strain’s metabolic profile reflects an individual metabolic ability, and a profile of a biota represents a population capacity. The metabolic profile of a fermentative biota could be used to monitor microbial quality and fermentation process (Prihatna and Suwanto 2007). Thanks to the ability to produce a variety of biological enzymes and metabolically active substances, *Rhizopus* is widely used in the food fermentation industry. *R. arrhizus* has a high utilization rate of carbohydrates (Fig. 4), in accordance with its well-known high saccharification ability (Yao et al. 2018). By tracking the change of metabolic fingerprints, the fermentation process and the biological activity of *R. arrhizus* could be under control.

The present study on the metabolic fingerprint showed that *R. arrhizus* was diverse in carbon/nitrogen assimilation and its comparative genomics, population genetics and epigenomics should be further investigated. Chibucos et al. (2016) have performed an integrated genomic and transcriptomic study on pathogenic *Mucorales*, including *R. arrhizus*, however, they did not take account of natural and industrial-relevant isolates. Previous reports on the metabolism of carbon/nitrogen sources in *R. arrhizus* rarely focused on its population diversity (Ghosh and Ray 2014), although Prihatna and Suwanto (2007) found a distinct separation in metabolic fingerprints among geographical origins of *Rhizopus oligosporus*, a species related to *R. arrhizus*. In the present study, we observed variation of substrate utilization within *R. arrhizus*, however, these distinctions did neither fall along the lines of isolation source (ecology) nor genetic lineage (taxonomic varieties), suggesting that divergence in gross substrate utilization has not evolved rapidly. At present, *R. arrhizus* were divided into three varieties, namely var. *arrhizus*, var. *tonkinensis*, and var. *delemar* (Zheng et al. 2007; Liu et al. 2008; Dolatabadi et al. 2014), and supported by some biochemistry and physiology studies (Londoño-Hernández et al. 2017; Saito et al. 2004; Yao et al. 2018). While two groups were categorized based on high utilization frequencies (HUFs), viz., 0–39.66% and 54.47–84.99% groups, which suggested *R. arrhizus* may have instead evolved to utilize a very broad and stable spectrum of carbon and nitrogen substrates.

This study identified several substrates that significantly promote the growth and sporangiospore production of *R. arrhizus* strains through high-throughput screening based on the Biolog FF MicroPlates, which containing 95 different carbon and nitrogen sources. The assimilation capabilities of *R. arrhizus* strains for different carbon and nitrogen sources were evaluated, revealing a certain substrate preference. Analyzing the assimilation capabilities of various *R. arrhizus* strains for carbon and nitrogen sources could be considered as a fingerprint for intra-specific variation. In summary, this study lays the foundation for the applications of *R. arrhizus* strains in fermentation and food industry, as well as provides new cultivation strategies to address strain degeneration.

### Supplementary Information

Supplementary material

## Data Availability

The experimental data support the findings of this study are available in Table 1 and Supplementary Materials.
