# Supplementary material for: High-throughput screening carbon and nitrogen sources to promote growth and sporulation in Rhizopus arrhizus
Source: AMB Express. 2024 Jun 28;14:76. doi: 10.1186/s13568-024-01733-0 (PMC11213844; doi:10.1186/s13568-024-01733-0)
Supplement: Supplementary file 1 — Supplementary material [file 13568_2024_1733_MOESM1_ESM.pdf]

# Supplemental Materials

**Journal name:** AMB Express

**Manuscript Title:** High-throughput screening carbon and nitrogen sources to promote growth and sporulation in *Rhizopus arrhizus*

Heng Zhao[1,2#], Xiao Ju[3,4#], Yong Nie[5], Timothy Y. James[6], Xiao-Yong Liu[1,3]*

[1] College of Life Sciences, Shandong Normal University, Jinan 250358, China.

[2] State Key Laboratory of Efficient Production of Forest Resources, School of Ecology and Nature Conservation, Beijing Forestry University, Beijing 100083, China. (zhaoheng181@mails.ucas.ac.cn)

[3] State Key Laboratory of Mycology, Institute of Microbiology, Chinese Academy of Sciences, Beijing 100101, China.

[4] Graduate School, China Pharmaceutical University, Nanjing 211198, China. (juxiao@cpu.edu.cn)

[5] School of Civil Engineering and Architecture, Anhui University of Technology, Ma'anshan, 243002, China. (nieyong@ahut.edu.cn)

[6] Department of Ecology and Evolutionary Biology, University of Michigan, Ann Arbor, MI 48109-1048, USA. (tyjames@umich.edu)

[#] These authors contributed equally to this work.

[*] Corresponding author: Xiao-Yong Liu, liuxy@sdnu.edu.cn

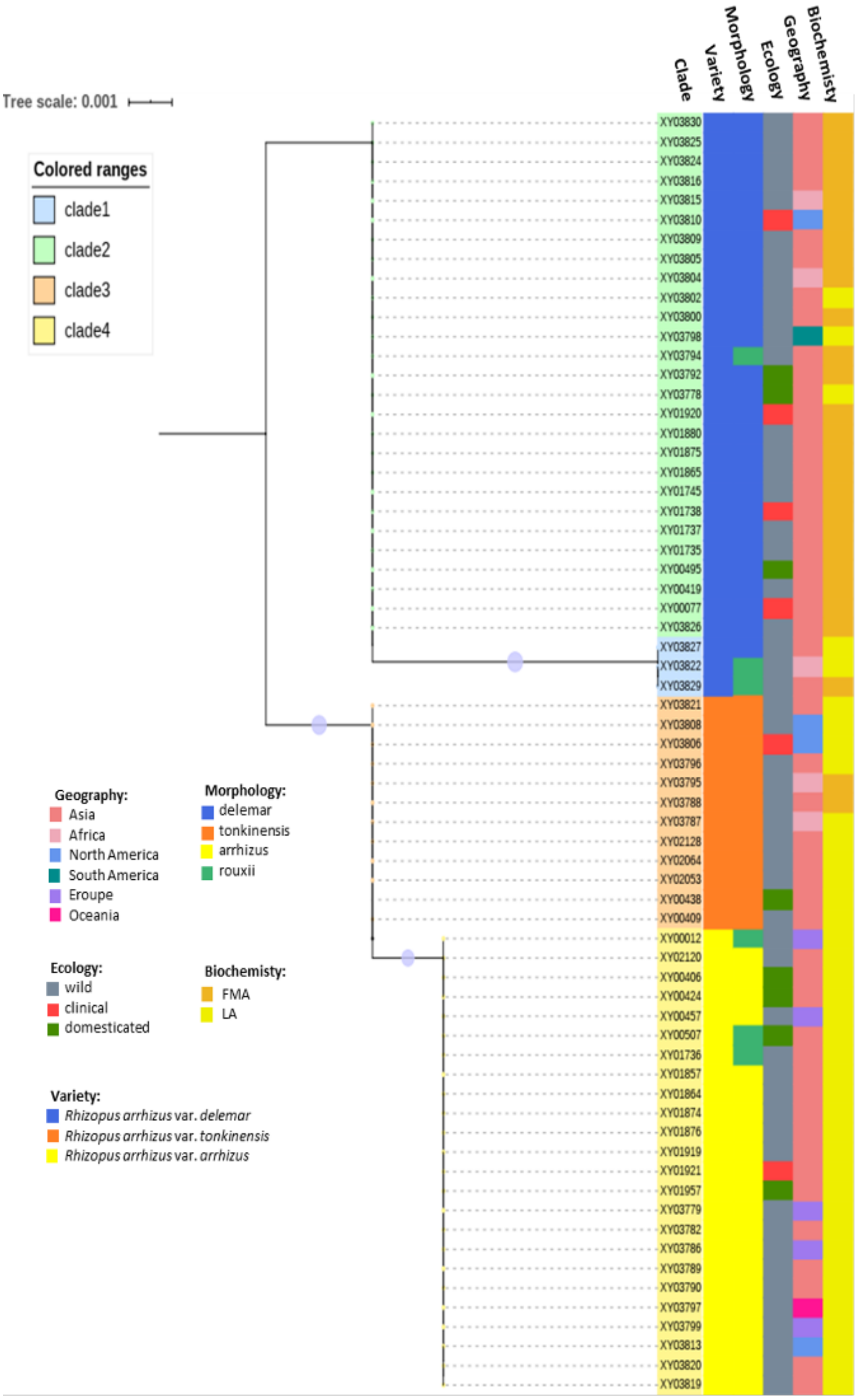


**Fig. S1.** Maximum likelihood phylogenetic tree based on ITS rDNA in *Rhizopus arrhizus*

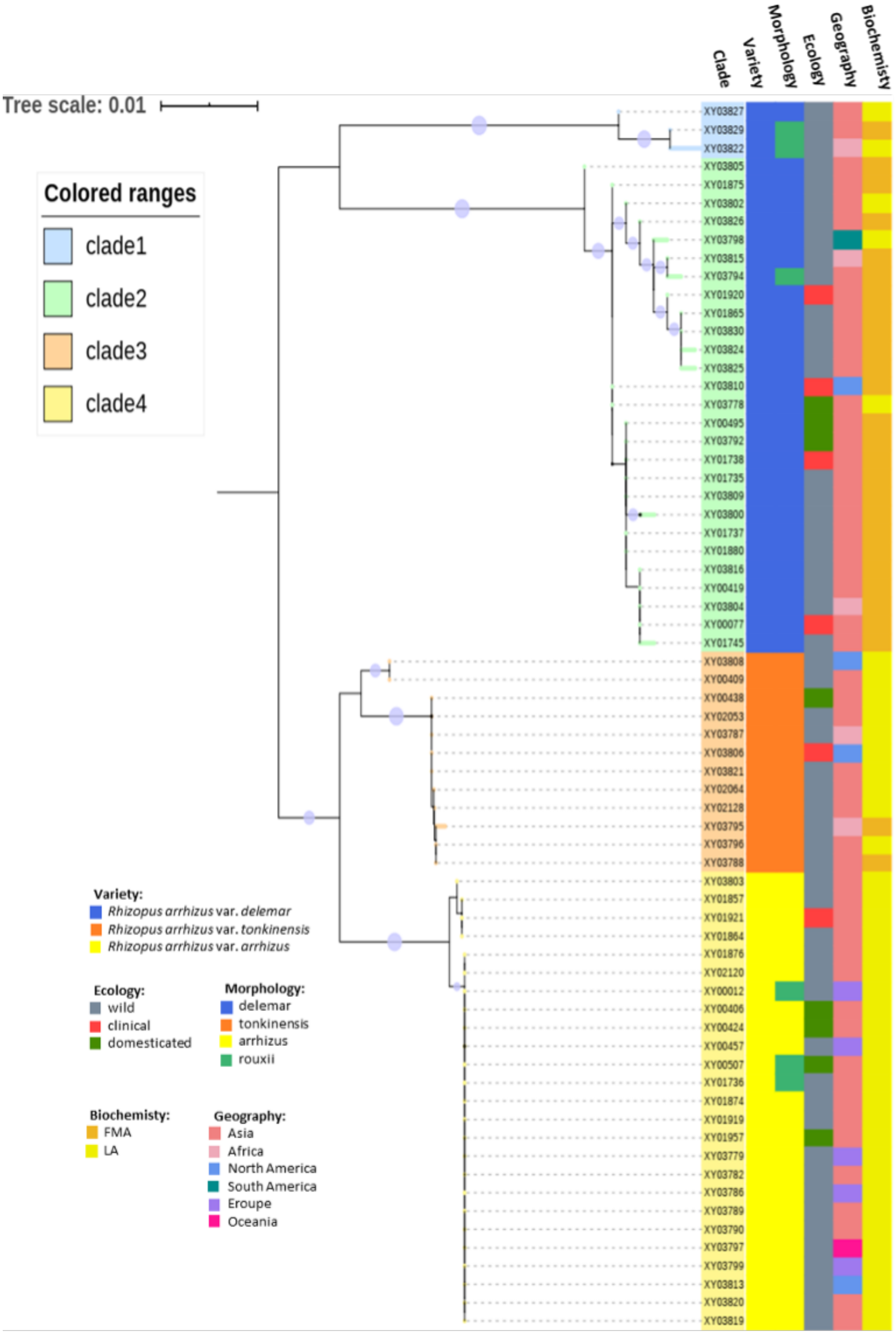


**Fig. S2.** Maximum likelihood phylogenetic tree based on IGS rDNA in *Rhizopus arrhizus*

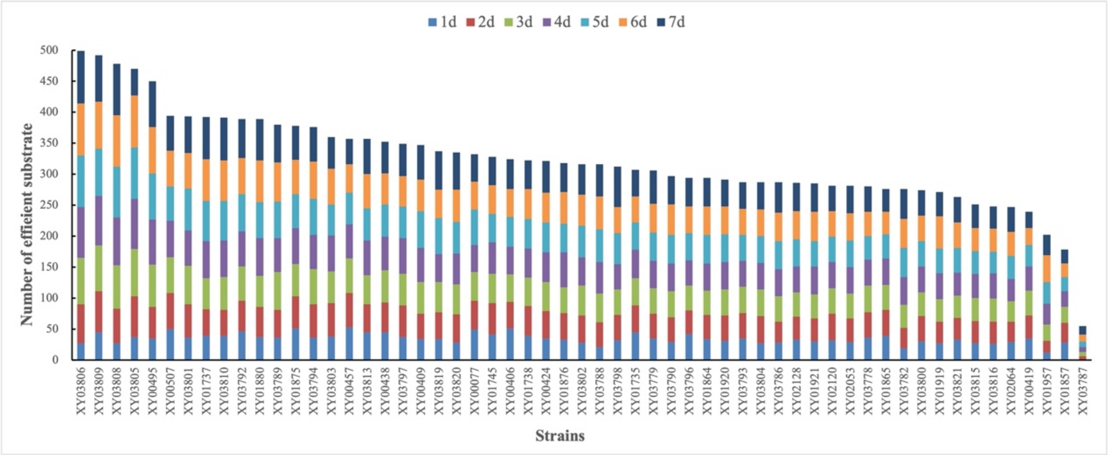


**Fig. S3.** The number of efficient substrates (y-axis) for 69 strains of *Rhizopus arrhizus* (x-axis) showing their substrate richness (SR) while being incubated with 95 carbon / nitrogen sources for seven days. Only those strains whose controlled and calibrated absorbance value is greater than 0.25 are counted. Absorbance values are means from three independent technical repeats each being read in triplicates

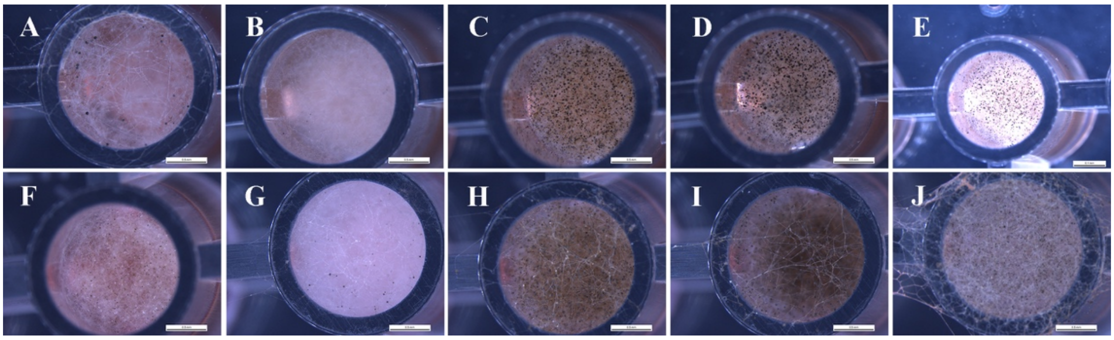


**Fig. S4.** The L-arabinose (A09) and xylitol (E11) rejuvenating degenerated *Rhizopus arrhizus* strains. A: XY00507 grown on L-arabinose, B: XY01736 grown on L-arabinose, C: XY03794 grown on L-arabinose, D: XY03822 grown on L-arabinose, E: XY03829 grown on L-arabinose, F: A: XY00507 grown on xylitol, G: XY01736 grown on xylitol, H: XY03794 grown on xylitol, I: XY03822 grown on xylitol, and J: XY03829 grown on xylitol.

**Table S1.** Information about substrates' ability of promoting the growth and sporulation of *Rhizopus arrhizus*.

| Wells Substrates | Sources | Categories | Representative strains for sporulation | | | | | Sporulation(%) | | Growth(%) | |
|---|---|---|---|---|---|---|---|---|---|---|---|
| | | | XY00507 | XY01736 | XY03794 | XY03822 | XY03829 | PF | HPF | UF | HUF |
| **A01:Water** | zero | zero | 0.00/0.00/– | 0.00/0.00/– | 0.00/0.00/– | 0.00/0.00/– | 0.00/0.00/– | 0.00 | 0.00 | 79.30 | 0.62 |
| **A02:Tween 80** | carbon | alcohol | 0.85/0.48/– | 0.82/0.40/+ | 0.55/0.46/+ | 0.53/0.45/+++ | 0.48/0.35/+ | 80.00 | 20.00 | 9.92 | 0.62 |
| **A03:N-acetyl-D-galactosamine** | nitrogen | amine | 0.22/0.02/– | 0.19/0.08/– | 0.31/0.11/– | 0.09/0.04/– | 0.07/0.02/– | 0.00 | 0.00 | 97.13 | 84.99 |
| **A04:N-acetyl-D-glucosamine** | nitrogen | amine | 2.09/1.4/++ | 2.02/1.38/+ | 2.33/1.68/+++ | 2.34/1.74/+++ | 2.46/1.94/+++ | 100.00 | 60.00 | 8.12 | 0.62 |
| **A05:N-acetyl-β-D-mannosamine** | nitrogen | amine | 0.06/0.02/– | 0.17/0.08/– | 0.19/0.06/– | 0.12/0.04/+ | -0.04/-0.02/– | 20.00 | 0.00 | 75.22 | 7.68 |
| **A06:Chunfushoucao** | carbon | alcohol | 1.25/0.99/++ | 1.94/1.79/+ | 1.05/1.14/+++ | 1.23/1.22/+++ | 1.99/1.93/+++ | 100.00 | 60.00 | 24.46 | 0.41 |
| **A07:Amygdalin** | carbon | glycoside | -0.23/0.1/– | 0.06/0.13/– | 0.73/1.02/++ | -0.20/0.11/+++ | -0.11/0.09/– | 40.00 | 20.00 | 37.07 | 0.62 |
| **A08:D-arabinose** | carbon | carbohydrate | 0.44/0.22/– | 0.4/0.17/+ | 0.33/0.26/– | 0.05/0.08/– | -0.26/-0.02/– | 20.00 | 0.00 | 68.50 | 13.04 |
| **A09:L-arabinose** | carbon | carbohydrate | 0.72/0.51/+ | 1.54/1.31/+ | 1.55/1.49/+++ | 1.40/1.40/+++ | 1.41/1.36/+++ | 100.00 | 60.00 | 84.71 | 14.65 |
| **A10:D-arabitol** | carbon | alcohol | 0.42/0.54/– | 0.82/0.67/– | 1.58/1.76/– | 1.43/1.47/+++ | 0.68/0.68/– | 20.00 | 20.00 | 87.76 | 2.28 |
| **A11:Arbutin** | carbon | glycoside | 0.45/0.42/– | 0.63/0.42/– | 1.17/1.49/++ | 0.89/1.09/+ | 0.41/0.35/– | 40.00 | 0.00 | 87.87 | 2.48 |
| **A12:D-cellobiose** | carbon | carbohydrate | 0.86/0.72/– | 0.80/0.69/– | 0.57/0.88/++ | 0.67/0.82/++ | 0.48/0.52/++ | 60.00 | 0.00 | 0.62 | 0.21 |
| **B01:α-cyclodextrin** | carbon | carbohydrate | -0.14/0.23/– | 0.04/0.17/– | -0.29/0.14/– | -0.32/0.10/– | -0.31/0.00/– | 0.00 | 0.00 | 33.02 | 0.62 |
| **B02:ß-cyclodextrin** | carbon | carbohydrate | -0.20/-0.07/– | -0.06/-0.04/++ | 0.93/0.85/++ | -0.02/-0.07/+ | -0.07/-0.11/+ | 80.00 | 0.00 | 87.90 | 0.62 |
| **B03:Dextrin** | carbon | carbohydrate | 1.01/0.60/– | 0.92/0.93/– | 0.90/0.91/+++ | 0.80/0.96/+++ | 0.60/0.78/+++ | 60.00 | 60.00 | 34.39 | 5.43 |
| **B04:I-erythritol** | carbon | alcohol | -0.32/0.04/– | -0.09/0.09/+ | -0.33/0.13/– | -0.37/0.06/– | -0.14/0.05/– | 20.00 | 0.00 | 93.76 | 4.67 |
| **B05:D-fructose** | carbon | carbohydrate | 0.80/0.67/– | 0.95/0.95/– | 1.22/1.47/++ | 1.44/1.48/+++ | 0.66/0.69/+++ | 60.00 | 40.00 | 3.30 | 0.21 |
| **B06:L-trehalose** | carbon | carbohydrate | 0.12/0.19/– | 0.08/0.12/+ | -0.13/0.05/– | -0.21/0.04/– | -0.08/0.07/– | 0.00 | 0.00 | 92.15 | 9.09 |
| **B07:D-galactose** | carbon | carbohydrate | 0.72/0.66/– | 0.94/0.74/+ | 0.70/0.76/– | 0.97/0.84/+ | 0.95/0.83/+ | 60.00 | 0.00 | 50.61 | 0.41 |
| **B08:D-galacturonic acid** | carbon | CAD | 0.08/0.40/+ | 0.51/0.54/++ | 0.37/0.57/– | 0.29/0.56/+ | 0.11/0.32/+ | 80.00 | 0.00 | 94.54 | 39.66 |
| **B09:Gentian disaccharide** | carbon | carbohydrate | 0.96/0.82/– | 1.56/1.46/– | 1.28/1.51/+++ | 1.94/1.92/+++ | 1.53/1.55/+++ | 60.00 | 60.00 | 80.27 | 4.63 |
| **B10:D-gluconic acid** | carbon | CAD | 1.36/1.22/– | 0.78/0.62/– | 0.89/0.73/++ | 0.24/0.14/+ | 0.30/0.19/+ | 60.00 | 0.00 | 36.54 | 1.31 |
| **B11:D-glucosamine** | carbon | amine | 0.35/0.44/– | 0.43/0.37/– | -0.02/0.30/– | 0.04/0.19/– | 0.16/0.17/– | 0.00 | 0.00 | 86.15 | 0.62 |
| **B12:α-D-glucose** | carbon | carbohydrate | 0.65/0.61/– | 0.81/0.80/– | 0.68/0.92/++ | 0.69/0.76/++ | 0.43/0.48/+ | 60.00 | 0.00 | 19.24 | 0.62 |
| **C01:α-D-glucose-1-phosphate** | carbon | carbohydrate | 0.17/0.01/– | -0.09/0.02/– | -0.17/0.04/– | -0.07/0.04/– | 0.1/0.04/– | 0.00 | 0.00 | 3.81 | 0.62 |
| **C02:Glucuromamide** | nitrogen | amine | -0.4/0.05/– | -0.10/0.06/– | -0.46/0.01/– | -0.41/0.03/– | -0.43/-0.05/– | 0.00 | 0.00 | 12.64 | 1.24 |
| **C03:D-glucuronic acid** | carbon | CAD | -0.48/0.01/– | -0.12/0.08/– | -0.38/0.09/– | -0.41/0.04/– | -0.32/0.02/– | 0.00 | 0.00 | 88.57 | 54.47 |
| **C04:Glycerin** | carbon | alcohol | 1.85/1.44/++ | 2.08/1.94/+ | 1.97/2.04/+++ | 1.85/1.81/+++ | 2.04/1.96/+++ | 100.00 | 60.00 | 87.99 | 0.62 |
| **C05:Hepatic sugar** | carbon | carbohydrate | 1.13/0.70/– | 0.76/0.62/– | 0.51/0.44/– | 0.77/0.67/++ | 0.37/0.24/+ | 40.00 | 0.00 | 18.93 | 1.51 |
| **C06:M-inositol** | carbon | carbohydrate | 0.22/0.07/– | 0.18/0.12/– | 0.25/0.23/– | -0.01/0.08/– | 0.04/0.05/– | 0.00 | 0.00 | 28.14 | 3.63 |
| **C07:2-keto-D-gluconic acid** | carbon | CAD | 0.35/0.15/– | 0.25/0.14/– | 0.36/0.21/– | -0.07/0.05/– | -0.02/0.06/– | 0.00 | 0.00 | 13.22 | 0.62 |
| **C08:α-D-lactose** | carbon | carbohydrate | 0.14/0.08/– | 0.18/0.11/– | 0.12/0.15/– | 0.13/0.10/– | 0.03/0.06/– | 0.00 | 0.00 | 12.66 | 1.07 |
| **C09:Lactulose** | carbon | carbohydrate | 0.06/0.06/– | 0.19/0.12/– | 0.10/0.16/– | 0.11/0.10/– | 0.07/0.07/– | 0.00 | 0.00 | 18.41 | 2.23 |
| **C10:Maltitol** | carbon | alcohol | 0.16/0.05/– | 0.23/0.13/– | 0.11/0.15/– | 0.17/0.12/– | 0.05/0.04/– | 0.00 | 0.00 | 94.82 | 23.46 |
| **C11:Maltose** | carbon | carbohydrate | 1.1/0.79/+ | 1.35/1.28/+ | 1.68/1.75/++ | 1.63/1.54/+++ | 1.22/1.07/++ | 100.00 | 20.00 | 89.75 | 1.69 |
| **C12: Rhamnose** | carbon | carbohydrate | 0.85/0.64/– | 0.81/0.70/+ | 1.28/1.42/– | 1.48/1.41/+++ | 0.81/0.67/+ | 60.00 | 20.00 | 87.51 | 23.06 |
| **D01:D-mannitol** | carbon | alcohol | 2.06/1.96/– | 2.02/1.88/+ | 1.95/1.89/+++ | 1.46/1.36/+++ | 1.15/1.05/+++ | 80.00 | 60.00 | 90.62 | 5.10 |
| **D02:D-mannose** | carbon | carbohydrate | 0.71/0.63/– | 1.18/1.20/+ | 0.79/1.01/++ | 1.18/1.17/+++ | 0.51/0.53/++ | 80.00 | 20.00 | 53.74 | 2.18 |
| **D03:D-pine triose** | carbon | carbohydrate | 0.34/0.09/– | 0.35/0.14/– | 0.38/0.18/– | 0.24/0.13/– | 0.19/0.06/– | 0.00 | 0.00 | 73.90 | 2.87 |
| **D04:D-disaccharide** | carbon | carbohydrate | 0.72/0.19/– | 0.78/0.25/+ | 0.67/0.41/++ | 0.87/0.64/++ | 1.06/0.77/++ | 80.00 | 0.00 | 42.51 | 0.62 |
| **D05:α-methyl-D-galactoside** | carbon | glycoside | 0.28/0.11/– | 0.35/0.19/– | 0.34/0.25/+++ | 0.32/0.2/++ | 0.75/0.53/++ | 60.00 | 20.00 | 22.42 | 0.62 |
| **D06:β-methyl-D-galactoside** | carbon | glycoside | 0.11/0.04/– | 0.12/0.11/– | 0.07/0.09/– | 0.14/0.07/– | 0.09/0.04/– | 0.00 | 0.00 | 10.27 | 1.10 |
| **D07:α-methyl-D-glucoside** | carbon | glycoside | 0.11/0.05/– | 0.07/0.05/– | 0.12/0.08/– | -0.01/0.07/– | -0.09/-0.08/– | 0.00 | 0.00 | 93.97 | 59.95 |
| **D08:β-methyl-D-glucoside** | carbon | glycoside | 1.26/0.83/+ | 1.80/1.80/– | 1.54/1.72/+++ | 1.88/1.94/– | 1.93/1.96/– | 40.00 | 20.00 | 19.09 | 2.67 |
| **D09:Isomaltulose** | carbon | carbohydrate | 0.17/0.08/– | 0.22/0.07/– | 0.00/0.12/– | -0.07/0.04/+++ | 0.01/0.07/+++ | 40.00 | 40.00 | 27.41 | 0.62 |
| **D10:D-allose** | carbon | carbohydrate | 0.25/0.18/– | 0.27/0.18/– | 0.15/0.21/– | -0.03/0.13/– | -0.08/0.08/– | 0.00 | 0.00 | 55.64 | 7.37 |
| **D11:D-honey triose** | carbon | carbohydrate | 0.32/0.10/– | 0.36/0.17/– | 1.15/1.16/+++ | 0.15/0.09/– | 1.02/0.81/++ | 40.00 | 20.00 | 10.11 | 1.31 |
| **D12:L-rhamnose** | carbon | carbohydrate | -0.33/0.01/– | -0.09/0.06/– | -0.39/0.00/– | -0.3/0.01/– | -0.14/0.01/ | 0.00 | 0.00 | 64.90 | 5.16 |
| **E01:D-ribose** | carbon | carbohydrate | 1.12/0.80/– | 1.26/1.17/++ | 1.39/1.27/+++ | 0.26/0.32/++ | 0.40/0.39/+ | 80.00 | 20.00 | 82.93 | 7.17 |
| **E02:Salicin** | carbon | glycoside | 0.35/0.27/– | 0.85/0.73/– | 1.07/1.33/– | 1.29/1.39/+ | 1.21/1.22/– | 20.00 | 0.00 | 2.13 | 0.86 |
| **E03:Sedoheptulosan** | carbon | carbohydrate | 0.03/0.05/– | 0.02/0.11/– | 0.04/0.13/– | -0.08/0.06/– | 0.01/0.06/– | 0.00 | 0.00 | 95.21 | 62.89 |
| **E04:D-sorbitol** | carbon | alcohol | 1.21/1.01/++ | 1.48/1.09/++ | 1.57/1.55/++ | 1.93/1.74/+++ | 1.17/1.17/++ | 100.00 | 20.00 | 10.09 | 4.59 |
| **E05:L-sorbose** | carbon | carbohydrate | -0.5/0.01/– | -0.06/0.13/– | -0.47/0.05/– | -0.39/0.08/– | -0.35/0.04/– | 0.00 | 0.00 | 42.74 | 8.57 |

| | | | | | | | | | | | |
|---|---|---|---|---|---|---|---|---|---|---|---|
| **E06:Stachyose** | carbon | carbohydrate | 0.09/0.02/– | 0.13/0.07/– | 1.10/0.90/++ | -0.34/0.17/– | 0.31/0.21/++ | 40.00 | 0.00 | 60.52 | 25.92 |
| **E07:Sucrose** | carbon | carbohydrate | 0.25/0.06/– | 0.04/0.05/– | 1.87/1.88/+++ | 0.12/0.06/+ | 1.25/1.28/+++ | 60.00 | 40.00 | 0.89 | 0.00 |
| **E08:D-tagatose** | carbon | carbohydrate | -0.39/0.04/– | -0.15/0.04/– | -0.32/0.03/– | -0.39/0.05/– | -0.29/0.03/– | 0.00 | 0.00 | 96.08 | 64.76 |
| **E09:D-trehalose** | carbon | carbohydrate | 1.78/1.33/++ | 1.82/1.64/+ | 2.22/2.05/+++ | 2.08/2.06/– | 2.13/2.03/+++ | 80.00 | 40.00 | 43.18 | 7.50 |
| **E10:Pine disaccharide** | carbon | carbohydrate | 0.26/0.02/– | 0.40/0.12/– | 0.24/0.15/– | 0.19/0.11/– | 0.16/0.08/– | 0.00 | 0.00 | 93.82 | 67.71 |
| **E11:Xylitol** | carbon | alcohol | 1.45/1.16/++ | 2.29/1.84/+ | 2.04/2.08/+++ | 2.00/1.95/+++ | 1.55/1.72/+++ | 100.00 | 60.00 | 92.58 | 19.25 |
| **E12:D-xylose** | carbon | carbohydrate | 1.05/0.76/– | 1.49/1.20/+ | 1.44/1.42/+ | 1.09/0.86/+++ | 1.14/0.93/++ | 80.00 | 20.00 | 82.26 | 22.17 |
| **F01:γ-aminobutyric acid** | nitrogen | amino acid | 1.51/0.96/– | 2.13/1.57/– | 1.55/1.27/+++ | 1.15/0.92/+++ | 1.71/1.41/+++ | 60.00 | 60.00 | 5.45 | 0.21 |
| **F02:Bromosuccinic acid** | carbon | CAD | -0.16/0.12/– | -0.08/0.11/– | -0.22/0.18/– | -0.32/0.11/– | -0.38/0.01/– | 0.00 | 0.00 | 36.15 | 0.83 |
| **F03:Fumaric acid** | carbon | CAD | 0.52/0.10/– | 0.56/0.07/– | 0.80/0.38/– | 0.23/0.20/– | 0.27/0.13/+ | 20.00 | 0.00 | 8.12 | 0.21 |
| **F04:β- hydroxybutyrate** | carbon | CAD | -0.26/0.03/– | -0.10/0.03/– | -0.10/0.03/– | -0.39/0.03/– | -0.19/0.02/– | 0.00 | 0.00 | 8.61 | 0.41 |
| **F05:γ-hydroxybutyric acid** | carbon | CAD | -0.31/0.00/– | -0.19/0.01/– | -0.29/0.03/– | -0.29/0.07/– | -0.22/0.06/– | 0.00 | 0.00 | 10.03 | 1.77 |
| **F06:P-hydroxyphenylacetic acid** | carbon | CAD | -0.38/0.04/– | -0.13/0.02/– | -0.43/0.02/– | -0.39/0.10/– | -0.33/0.02/– | 0.00 | 0.00 | 84.55 | 0.83 |
| **F07:Alpha-ketoglutaric acid** | carbon | CAD | 0.62/0.11/– | 0.54/0.10/– | 0.53/0.24/– | 0.55/0.19/– | 0.33/0.09/– | 0.00 | 0.00 | 14.52 | 0.41 |
| **F08:D-methyl lactate** | carbon | CAD | 0.53/0.07/– | 0.24/0.05/– | 0.77/0.06/– | -0.22/0.08/– | -0.01/0.05/– | 0.00 | 0.00 | 29.70 | 0.62 |
| **F09:L-lactic acid** | carbon | CAD | 0.33/0.10/– | 0.41/0.09/– | 0.30/0.09/– | 0.31/0.14/– | 0.02/0.06/– | 0.00 | 0.00 | 18.81 | 0.62 |
| **F10:D-malic acid** | carbon | CAD | 0.42/0.11/– | 0.35/0.12/– | -0.02/0.13/– | 0.01/0.09/– | 0.06/0.09/– | 0.00 | 0.00 | 52.45 | 0.62 |
| **F11:L-malic acid** | carbon | CAD | 0.52/0.10/– | 0.69/0.16/– | 0.44/0.26/– | 0.42/0.15/– | 0.38/0.11/– | 0.00 | 0.00 | 15.61 | 2.79 |
| **F12:Quinic acid** | carbon | CAD | 0.17/0.03/– | 0.08/0.06/– | -0.03/-0.01/– | -0.03/0.03/– | -0.01/0.01/– | 0.00 | 0.00 | 9.92 | 0.41 |
| **G01:D-gluconic acid** | carbon | CAD | -0.19/-0.01/– | -0.13/0.01/– | -0.21/-0.02/– | -0.13/0.01/– | -0.2/0.01/– | 0.00 | 0.00 | 18.42 | 0.62 |
| **G02:Sebacic acid** | carbon | CAD | 0.57/0.82/+ | 0.7/0.88/+ | 0.27/0.62/+++ | 0.26/0.56/+++ | -0.32/0.01/++ | 100.00 | 40.00 | 12.44 | 1.75 |
| **G03:Succinamic acid** | carbon | CAD | 0.43/0.16/– | -0.05/0.08/– | 0.24/0.06/– | -0.34/0.05/- | -0.27/0.07/- | 0.00 | 0.00 | 48.60 | 0.62 |
| **G04:Succinic acid** | carbon | CAD | 0.52/0.16/– | 0.69/0.16/– | 0.51/0.46/– | 0.57/0.39/+ | 0.45/0.23/+ | 40.00 | 0.00 | 14.71 | 0.41 |
| **G05:Monomethyl succinate** | carbon | CAD | 0.25/0.25/– | -0.01/0.10/– | -0.16/0.07/– | -0.39/0.07/– | -0.31/0.06/– | 0.00 | 0.00 | 6.40 | 0.21 |
| **G06:N-acetyl-L glutamic acid** | nitrogen | amino acid | -0.03/0.07/– | -0.04/0.03/– | 0.12/0.10/– | -0.35/0.15/– | -0.16/0.05/– | 0.00 | 0.00 | 69.40 | 0.62 |
| **G07:L-alanamine** | nitrogen | CAD | 0.57/0.20/– | 0.32/0.07/– | 0.56/0.14/– | 0.37/0.13/– | 0.33/0.14/– | 0.00 | 0.00 | 95.69 | 29.83 |
| **G08:L-alanine** | nitrogen | amino acid | 0.97/0.43/– | 1.13/0.40/– | 1.71/1.09/+++ | 1.40/0.83/++ | 1.23/0.79/++ | 60.00 | 20.00 | 75.56 | 1.96 |
| **G09:L-alanyl-glycine** | nitrogen | amino acid | 0.77/0.22/– | 0.51/0.10/– | 1.02/0.50/– | 0.74/0.33/+ | 0.54/0.25/+ | 40.00 | 0.00 | 96.04 | 14.81 |
| **G10:L-asparagine** | nitrogen | CAD | 0.83/0.26/– | 1.03/0.36/– | 1.71/1.02/+ | 1.36/0.58/+ | 0.91/0.51/+ | 60.00 | 0.00 | 91.18 | 0.62 |
| **G11:L-aspartate** | nitrogen | amino acid | 0.64/0.19/– | 0.88/0.23/– | 1.03/0.41/– | 0.83/0.31/+ | 0.70/0.39/+ | 40.00 | 0.00 | 92.49 | 5.32 |
| **G12:L-glutamic acid** | nitrogen | amino acid | 0.72/0.31/– | 1.03/0.38/– | 1.28/0.76/+ | 1.50/0.98/++ | 0.88/0.60/++ | 60.00 | 0.00 | 16.61 | 0.62 |
| **H01:Gglycyl-L-glutamic acid** | nitrogen | amino acid | 0.69/0.08/– | 0.25/0.01/– | -0.11/0.00/– | 0.03/-0.02/– | -0.19/0.00/– | 0.00 | 0.00 | 96.93 | 58.52 |
| **H02:L-ornithine** | nitrogen | amino acid | 1.06/0.54/– | 1.50/0.84/– | 1.85/1.17/+ | 1.20/0.64/++ | 1.18/0.76/++ | 60.00 | 0.00 | 14.76 | 1.32 |
| **H03:L-phenylalanine** | nitrogen | amino acid | -0.17/-0.06/– | -0.21/-0.04/– | -0.30/-0.01/– | -0.24/0.06/– | -0.09/-0.01/– | 0.00 | 0.00 | 95.41 | 75.46 |
| **H04:L-valine** | nitrogen | amino acid | 1.70/1.41/– | 1.68/1.16/+ | 2.17/1.55/+ | 1.86/1.48/+++ | 1.99/1.58/+++ | 80.00 | 40.00 | 16.22 | 2.93 |
| **H05:L-pyroglutamic acid** | nitrogen | amino acid | 0.27/0.01/– | -0.11/-0.01/– | 0.24/0.03/– | -0.34/0.02/– | -0.18/0.05/– | 0.00 | 0.00 | 55.36 | 1.32 |
| **H06:L-serine** | nitrogen | amino acid | 0.73/0.15/– | -0.11/0.01/– | 0.03/0.17/+ | -0.45/0.04/ | -0.15/0.02/++ | 40.00 | 0.00 | 18.68 | 0.84 |
| **H07:L-threonine** | nitrogen | amino acid | -0.11/0.01/– | -0.21/-0.01/– | -0.26/-0.01/– | -0.18/0.03/– | -0.20/-0.01/– | 0.00 | 0.00 | 7.37 | 0.21 |
| **H08:2-aminoethanol** | carbon | alcohol | -0.54/-0.06/– | -0.27/-0.05/– | -0.57/-0.06/– | -0.46/0.00/– | -0.34/-0.02/– | 0.00 | 0.00 | 75.89 | 1.04 |
| **H09:Putrescine** | nitrogen | amine | 0.73/0.53/– | 0.86/0.53/– | 0.38/0.24/+++ | 0.67/0.69/+++ | 0.94/0.89/+++ | 60.00 | 60.00 | 0.41 | 0.00 |
| **H10:Adenosine** | carbon | glycoside | -0.9/-0.46/– | -0.73/-0.48/– | -0.60/-0.30/– | -0.75/-0.37/– | -0.96/-0.59/– | 0.00 | 0.00 | 4.51 | 0.62 |
| **H11:Guanosine** | carbon | glycoside | -0.07/-0.01/– | -0.19/0.00/– | -0.19/0.04/– | -0.08/0.07/– | -0.09/0.03/– | 0.00 | 0.00 | 3.28 | 0.21 |
| **H12:5-monophosphate adenosine** | carbon | glycoside | -0.22/-0.005/– | -0.12/0.05/– | -0.37/-0.01/– | -0.30/-0.02/– | -0.26/-0.01/– | 0.00 | 0.00 | 79.30 | 0.62 |

Note: The codes of wells in the Bilog FF MicroPlate are followed by the name of substrates, and they are separated with a colon. Absorbance, turbidity and sporulation of five selected *Rhizopus arrhizus* strains while being incubated with Biolog FF MicroPlate and divided with a slash "/". "CAD" is short for carboxylic acids and derivatives. The symbols “–” “+” “++”and “+++” mean no, poor, moderate and heavy sporulation, respectively. "PF" and "HPF" are abbreviated for promotion frequency and high promotion frequency, respectively, and relevant values are calculated from the five selected strains. "UF" and "HUF" are abbreviated for utilization frequency and high utilization frequency, respectively, and relevant values are averages of seven-day utilization frequencies. PF=100%, HPF=60%, UF≥95% and HUF≥75% are shaded.

**Table S2.** Thirteen special strains of *Rhizopus arrhizus* revealed by AWCD, SR, PCA and heatmapping.

| Strains | AWCD | SR | PCA | Heatmap |
|---|---|---|---|---|
| XY00077 | | | | yes |
| XY00495 | | yes | | yes |
| XY01874 | | yes | | |
| XY03787 | yes | yes | yes | |
| XY03799 | | | | yes |
| XY03801 | | | | yes |
| XY03802 | | | yes | |
| XY03805 | yes | yes | | yes |
| XY03806 | yes | yes | yes | yes |
| XY03808 | yes | yes | | |
| XY03809 | yes | yes | | yes |
| XY03813 | | | | yes |
| XY03827 | yes | yes | | yes |
